# Pain and Disability Therapy with Stabilization Exercises in Patients with Chronic Low Back Pain: A Meta-Analysis

**DOI:** 10.3390/healthcare13090960

**Published:** 2025-04-22

**Authors:** Vanja Dimitrijević, Bojan Rašković, Nikola Jevtić, Siniša Nikolić, Dejan Viduka, Borislav Obradović

**Affiliations:** 1 Faculty of Sports and Physical Education, University of Novi Sad, 21000 Novi Sad, Serbia; dimitrijevicvanja@gmail.com (V.D.); boriscons@yahoo.com (B.O.); 2 Performane Zone, 21000 Novi Sad, Serbia; 3 Scolio Centar, 21000 Novi Sad, Serbia; njevticns@gmail.com; 4 Institute of Physical Medicine, Rehabilitation and Orthopedic Surgery “Dr. Miroslav Zotović”, 78000 Banja Luka, Bosnia and Herzegovina; sinisamnikolicbl@gmail.com; 5 Faculty of Information Technologies, University Alfa BK, 11000 Belgrade, Serbia; dejan.viduka@alfa.edu.rs

**Keywords:** low back pain, back pain, conservative treatment, exercise therapy

## Abstract

Background: Chronic low back pain is a leading cause of disability worldwide, necessitating effective interventions to alleviate pain and improve function. This meta-analysis aimed to evaluate the effectiveness of stabilization exercises for pain relief and disability reduction in patients with chronic low back pain. Methods: A meta-analysis was conducted following PRISMA and Cochrane guidelines. Randomized controlled trials evaluating stabilization exercises for chronic low back pain were included. Subgroup analyses were performed based on treatment duration, type of pain (specific vs. non-specific), study quality, and exercise type. Effect sizes were calculated using standardized mean differences (SMD), and evidence quality was assessed using the GRADE tool. Results: A total of 23 studies involving 1132 participants were included. The meta-analysis revealed that longer treatment durations (8–12 weeks) showed the strongest effects on pain reduction (SMD = −0.88) and disability improvement (SMD = −0.85). For pain type, non-specific low back pain responded better (SMD = −0.81 for pain, −0.73 for disability) compared to specific LBP (SMD = −0.61 and −0.42, respectively). The 6-week duration also demonstrated moderate effects (SMD = −0.72 for pain). Core stability exercises had superior pain reduction (SMD = −0.90, large effect) compared to spinal stability exercises (SMD = −0.57), while spinal stability exercises showed higher-quality evidence for disability improvement (SMD = −0.56, high-quality) versus core stability (SMD = −0.62, low-quality). Conclusion: Stabilization exercises are a highly effective intervention for chronic low back pain, offering significant pain relief and functional improvement. They outperform other common interventions and should be prioritized in clinical practice, particularly in longer-duration, supervised programs. These findings provide strong evidence to guide treatment protocols and improve outcomes for patients with chronic low back pain.

## 1. Introduction

Low back pain (LBP) represents a global health crisis as the leading cause of disability worldwide [1], with the lumbar region affected in 80% of cases [2]. The condition imposes a substantial economic burden through direct healthcare costs and indirect productivity losses [3], while epidemiological studies confirm that 50–80% of adults will experience LBP at some point in their lives [4,5,6,7,8]. The Lancet Low Back Pain Series underscores that LBP is the leading cause of disability worldwide, with rising prevalence and inadequate healthcare responses [1,9]. Recent analyses highlight that up to 60% of LBP care is “low-value”, exacerbating costs and patient outcomes [3,10]. Definitions of LBP vary, but it is generally described as pain or discomfort located between the lower ribs and the upper thighs, sometimes accompanied by leg pain [11,12]. The study by [13] emphasizes the need to shift from a purely biomedical model to a biopsychosocial approach, given the complexity of LBP. LBP is classified by duration (acute/subacute/chronic) [11,12] and etiology (specific vs. non-specific), with 10% of non-specific cases becoming chronic [14,15,16]. Chronic low back pain (CLBP) lasts 12 weeks and can be classified based on etiology, pain mechanisms, and clinical presentation [17]. Despite its widespread impact, many individuals with LBP do not seek medical care, and those who do often report similar pain levels and frequency as those who do not [18,19,20]. Recent studies highlight structural and functional changes in back muscles among individuals with LBP, including atrophy of the multifidus and altered motor control [21,22].

Evidence strongly supports the effectiveness of exercise and multidisciplinary pain management programs in treating LBP [23,24]. The study by [9] calls for prioritizing non-pharmacological interventions, particularly exercise therapy, as first-line treatment. A network meta-analysis found that motor control and stabilization exercises are among the most effective modalities for managing chronic LBP [25]. Common exercises for LBP include warm-ups, strengthening exercises for the back extensors, abdominals, glutes, and leg muscles, as well as flexibility routines [26]. Hamstring tightness can negatively impact lumbopelvic biomechanics, making stretching exercises a valuable component of LBP treatment [27,28]. Lumbar, spinal, and trunk stabilization exercises are key rehabilitation strategies for managing LBP by improving stability and muscle coordination and reducing excessive spinal movement [29]. These exercises target the deep stabilizing muscles, including the transversus abdominis, multifidus, diaphragm, and pelvic floor muscles [30]. The study by [31] confirms that motor control exercises significantly reduce pain and disability in chronic LBP. Core stability exercises aim to strengthen neuromuscular and motor control systems, reducing the risk of spinal injuries [32].

Our PICOS question is as follows: In patients with chronic low back pain (P), does stabilization exercise therapy (I) compared with other treatments (C) lead to a reduction in pain and improvement in functional status (O), analyzed through randomized controlled trials (S)? The primary objective of this study is to systematically evaluate the efficacy of stabilization exercises in reducing pain and improving functional outcomes in patients with chronic low back pain through a comprehensive meta-analysis of randomized controlled trials. This analysis will be based on standardized outcome measures to ensure consistency and comparability across studies. The secondary objectives are to compare the relative effectiveness of different stabilization exercise approaches—such as core stabilization versus spinal stability exercises—and to assess the impact of varying treatment durations. These secondary aims seek to establish clinically meaningful, evidence-based recommendations for rehabilitation protocols, thereby supporting more informed clinical decision-making in managing chronic low back pain.

## 2. Materials and Methods

This paper was developed and reported according to the Preferred Reporting Items for Systematic Reviews and Meta-Analyses (PRISMA) guidelines [33] and the Cochrane guidelines [34], and it was registered in the PROSPERO database (CRD42022371282).

### 2.1. Deviations from the Protocol

After the first search, which was based on the outcome of quality of life, studies with various types of treatments were obtained, so a precise differentiation of these treatments could not be made based on them. In the second search, we based ourselves on the outcomes of pain and disability, which were secondary to us in the first search. Before the first search, we did not define a specific conservative treatment we would use in the analysis. For the basic treatment in the second search, we used stabilization exercises.

### 2.2. Inclusion and Exclusion Criteria

**Figure 1:**
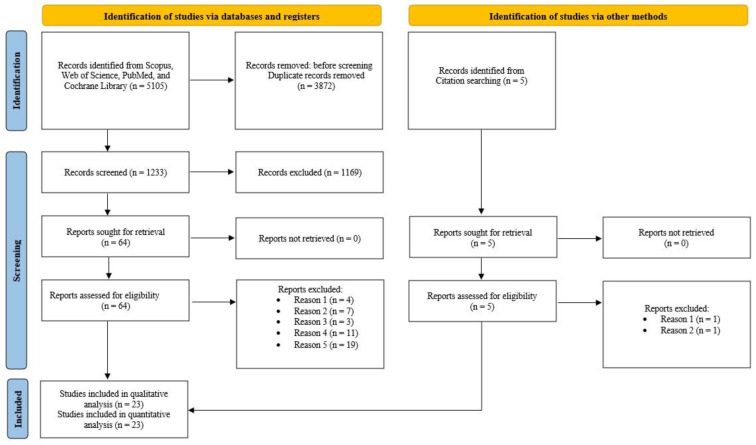
Flow chart.

A search strategy was developed to identify all relevant studies evaluating the effect of different methods in the treatment of CLBP. Our systematic search included PubMed, Cochrane Library, Web of Science, and Scopus databases. We used combinations of subject headings, adapting them to each database: (“Low back pain” OR “Back pain” OR “LBP” OR “Lumbago” OR “Lumb pain” OR “Backache”), AND (“Conservative treatment” OR “Exercise therapy” OR “Stabilization exercise” OR “Core stabilization” OR “Physical exercise” OR “Lumbar stabilization” OR “Trunk stabilization”) The search was conducted on 11 January 2025. Figure 1 shows the search strategy.

PICOS (population, interventions, comparators, outcomes, study designs) eligibility criteria described in PRISMA were adopted for inclusion/exclusion of the studies (Table 1) [35].

### 2.3. Information Sources, Selection, and Data Collection Process

Four databases were searched (PubMed, Cochrane Library, Web of Science, and Scopus). Two investigators, B.R. and V.D., included/excluded studies. In case of disagreement, the problem is solved by agreement and consensus. Two investigators independently performed data extraction after selecting studies based on all inclusion and exclusion criteria in the meta-analysis, presented in the PRISMA flow diagram (Figure 1). Mendeley Desktop (version 1.19.8, Elsevier) was used for reference management and citation organization. All identified studies from the databases were imported into Mendeley, where duplicate records were removed. After screening and selecting the eligible studies, all relevant data were extracted and transferred to Microsoft Excel (2016) for further analysis. The following variables are tabulated: authors, year of publication, program type, number of participants, age, outcomes, sessions per week, duration, and country (Table 2).

### 2.4. Data Analysis

Meta-analysis and statistical analysis were performed using R 4.3.2 software with the meta package [36]. Effect sizes were estimated for pain and disability outcomes. For each study, standardized mean difference (SMD) and 95% confidence intervals (CI) were calculated for continuous outcomes, random model. According to Cohen’s guide, values of 0.2–0.5, 0.5–0.8, and >0.8 show small, medium, and large effect sizes, respectively [37]. Results were considered statistically significant at *p* < 0.05. After the basic analyses, subgroup analyses were performed for the factors of duration of treatment, type of pain, risk of bias, and differences in the type of treatment. In our meta-analysis, we included both a spinal stabilization group and a core stabilization group to explore potential differences in their effects on CLBP. Although many exercises overlap between these two approaches, we performed a subgroup analysis to account for the differentiation made by the authors of the included studies, as reflected in the naming and categorization of their exercises. This subgroup analysis allows us to better understand whether the specific focus of the interventions—spinal stabilization versus core stabilization—may influence outcomes, even if the exercises themselves share similarities. By doing so, we aim to provide a more nuanced interpretation of the results and acknowledge the distinctions emphasized in the original studies. The subgroup of spinal stability exercises is composed of lumbar, trunk, and spinal stability exercises, as defined by the authors of the included studies. Heterogeneity was assessed using the Higgins I^2^ test [38] and *p* values, and Egger’s test [39] investigated publication bias.

### 2.5. Quality Assessment and Quality of Evidence

Two investigators independently assessed the quality of the studies. The risk of bias was assessed for each randomized trial using the Cochrane Risk of Bias Tool [34], which assesses seven sources of bias. Each study was examined and rated as low, high, or unclear. The quality of meta-analysis records was evaluated using the GRADE (Grading of Recommendations, Assessment, Development, and Evaluation) tool [40]. Evidence quality was categorized as “High”, “Moderate”, “Low”, or “Very Low”, reflecting the level of confidence that the true effect size aligns closely with the observed effect size. Several factors contributed to a downgrade in quality, including the risk of bias, inconsistency, indirectness, imprecision, and publication bias. The risk of bias led to a one-level downgrade if it was likely to significantly alter the results, particularly when studies included a mix of low and moderate risk of bias. Each study’s overall risk of bias was classified as “Low”, “Moderate”, or “High”. If a substantial portion of the data came from high-risk studies, the quality was reduced by one level for serious risk or two for very serious risk. Inconsistency was considered high when there was significant variability in effect sizes across studies, minimal overlap in confidence intervals, or strong statistical evidence of heterogeneity. Indirectness was a concern if the studies had limitations related to the population, intervention, comparator, or outcomes. Imprecision resulted in a downgrade if the total sample size was below 400 or if the confidence interval’s lower or upper limit exceeded 0.5 of the standardized mean difference (SMD) in either direction. Publication bias was assessed based on asymmetry in the meta-analysis funnel (with at least 10 included studies), underreporting of negative findings, or reported conflicts of interest among researchers [41].

## 3. Results

Based on the search strategy, a total of 665 studies were selected from the initial database search. Of that number, 198 duplicate studies were first excluded; therefore, 467 studies were selected for further analysis. A total of 388 studies were excluded after screening the abstracts and titles because they did not meet the inclusion criteria. The remaining 64 studies were fully reviewed. Following a comprehensive full-text assessment, 44 studies were deemed ineligible and subsequently excluded. The remaining 20 studies that met all criteria were included in this review article and meta-analysis. Three more studies that met the inclusion criteria were found through a citation search. The flow diagram is shown in Figure 1.

Table 2 shows the characteristics of the included studies. A total of 1132 respondents participated in 23 studies; the sample ranged from 19 to 160, while the respondents were 18 years of age and older. The total length of treatment ranged from 2 weeks to 12 weeks. The study analyzed the effect of treatment on pain reduction using a sample of 796 participants, while its impact on disability was evaluated using a sample of 782 participants.

Figures S1 and S2 (Supplementary Materials) show the risk of bias. Of the 23 included studies, all were randomized. Concealment of allocation was high risk in 14 out of 23 randomized studies. Physiotherapists and participants could not be blinded due to the way the intervention was applied, so all randomized studies were assessed as unclear risk. For the outcome “Blinding of outcome assessment”, ten studies had a low risk. The studies by [42,43] present data in a manner inappropriate for meta-analysis, which poses a problem in data processing, and it is this item that is assessed as high risk. Of the 161 items in the 23 included randomized studies, there were 29 high items (18.01%), 24 unclear items (14.9%), and 108 low items (67.08%). To see how studies with different quality affect the overall effect sizes in all measured outcomes, subgroup analyses were performed, the results of which are presented in the results of the meta-analysis.

**Table 2:** Main features of the included studies.

Study	Type of Exercise	N	Age	Outcomes	Exercise Time	Duration	Type of	Country
	Program				Per Week		LBP	
Akhtar [44]	Core stability exercises vs.	108	46.39 ± 7.43	VAS	N/A	6 weeks	Non-specific	Pakistan
	Physical Exercise		45.50 ± 6.61					
Alfuth [45]	Core stability exercises vs.	27	43 ± 9.2	RM	N/A	4 weeks	Specific	Deutschland
	Mobilization exercises		50 ± 8.5	ODQ				
Bae [46]	Core stability exercises vs.	36	32.4 ± 10.7	VAS	3 × 30 min	4 weeks	Non-specific	Republic of Korea
	Sit-up exercise		32.7 ± 6.1	ODQ				
				RM				
Bhadauria [47]	Lumbar stability exercises vs.	24	32.75 ± 11.73	VAS	3–4 × 60 min	3 weeks	Specific	India
	Dynamic Strengthening		36.67 ± 10.74	ODQ				
	Pilates		35.33 ± 12.88					
Gorji [48]	Core stability exercises vs.	37	54.6 ± 2.4	VAS	3 × 45–60 min	8 weeks	Specific	Iran
	PNE/MCE		55.2 ± 2.6	RM				
Hosseinifar [49]	Lumbar stability exercises vs.	30	34.2 ± 7.1	VAS	3 × 45 min	6 weeks	Non-specific	Iran
	McKenzie Exercises		33.8 ± 6.9					
Hwangbo [50]	Core stability exercises vs.	30	46.7 ± 10.2	VAS	3 × 60 min	6 weeks	Specific	Republic of Korea
	Combined exercise program		47.3 ± 9.8					
Javadian [51]	Lumbar stability exercises	30	38.5 ± 9.2	VAS	3 × 45 min	8 weeks	Non-specific	Iran
	Conventional physiotherapy		37.8 ± 8.7	ODQ				
Karimi [43]	Core stability exercises	29	42.3 ± 10.5	VAS	3 × 60 min	6 weeks	Non-specific	Iran
	Standard care		41.8 ± 9.7	ODQ				
Ko [52]	Lumbar stability exercises	19	45.2 ± 11.3	VAS	3 × 60 min	12 weeks	Specific	Republic of Korea
	Conventional physiotherapy		44.8 ± 10.7					
Kofotolis [53]	Spinal stability exercises	44	41.0 ± 5.5	BRP	3 × 45 min	4 weeks	Specific	Greece
	Standard care		42.2 ± 7.8	ODQ				
Koumantakis [54]	Spinal stability exercises	55	39.2 ± 11.4	VAS	2 × 60 min	8 weeks	Specific	Greece
	General exercise		35.2 ± 9.7	RM				
Lee [55]	Lumbar stability exercises	40	34.75 ± 0.85	VAS	4 × 60 min	6 weeks	Specific	Republic of Korea
	Conventional physiotherapy		34.20 ± 0.69					
Lee [56]	Lumbar stability exercises	39	54.9 ± 10.6	ODQ	3 × 20 min	6 weeks	Non-specific	Republic of Korea
	Resistance hip exercises		50.0 ± 11.4					
Nabavi [42]	Trunk stability exercises	41	40.75 ± 8.23	VAS	3 × 45 min	4 weeks	Non-specific	Iran
	Conventional physiotherapy		34.05 ± 10.75					
Noormohammadpour [57]	Core stability exercises	20	43.3 ± 7.5	VAS	3 × 60 min	8 weeks	Non-specific	Iran
	Conventional physiotherapy		41.3 ± 6.4	RM				
Puntumetakul [58]	Core stability exercises	38	37.26 ± 13.38	NRS	2 × 20 min	10 weeks	Specific	Thailand
	Strengthening exercise		39.10 ± 10.91					
Salavati [59]	Trunk stability exercises	40	32.60 ± 7.80	VAS	3 × 45 min	4 weeks	Non-specific	Iran
	General exercise		29.93 ± 5.18	ODQ				
Shaughnessy [60]	Lumbar stability exercises	41	43 ± 9	RM	2 × 30 min	10 weeks	Specific	Ireland
	Standard care		46 ± 11	ODQ				
Stankovic [61]	Lumbar stability exercises	160	49.5 ± 11.8	VAS	3 × 60 min	4 weeks	Specific	Serbia
	Strengthening and stretching		49.5 ± 12.4	ODQ				
Suh [62]	Lumbar stability exercises	23	57.40 ± 15.88	VAS	3 × 60 min	6 weeks	Specific	Republic of Korea
	Walking exercise		54.15 ± 13.89	ODQ				
Ulger [63]	Trunk stability exercises	113	41.6 ± 12.9	VAS	N/A	6 weeks	Specific	Turkey
	Manual therapy		43.1 ± 14.3	ODQ				
Waseem [64]	Core stability exercises	108	46.39 ± 7.43	ODQ	3 × 45 min	6 weeks	Non-specific	Pakistan
	Conventional physiotherapy		45.50 ± 6.61					

LBP—low back pain; VAS—visual analogue scale; ODQ—Oswestry disability questionnaire; RM—Roland–Morris disability questionnaire; NRS—numeric rating scale; BRP—Borg verbal rating pain scale; PNE—pain neuroscience education; MCE—motor control exercises.

Meta-analysis

### 3.1. The Influence of the Duration of the Treatment on the Pain

Short duration

**Figure 2:**
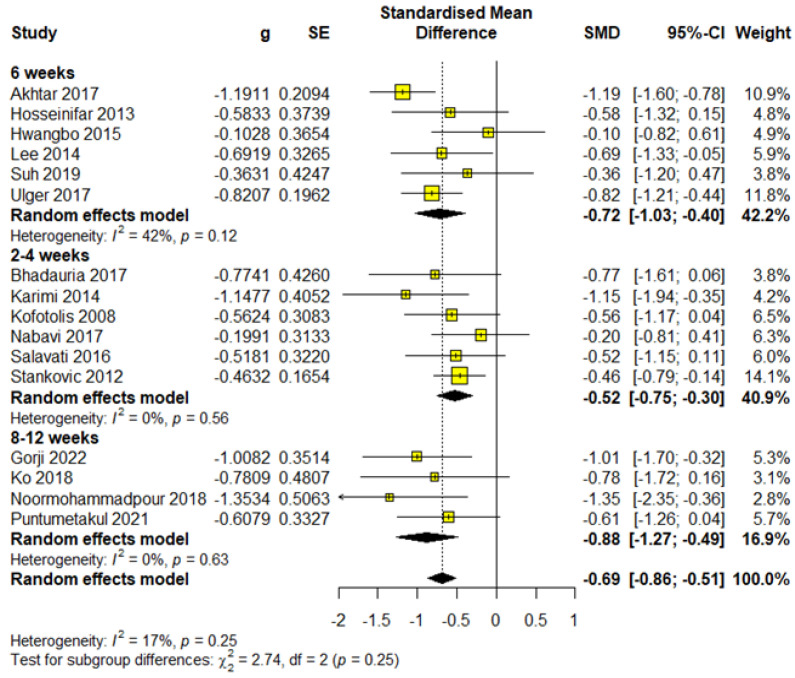
Forest plot for outcome pain—subgroup treatment duration.

The shortest treatment duration was categorized within the 2- to 4-week subgroup. A total of six studies utilized this treatment length, involving 338 participants, which accounted for 42.46% of the total sample. Meta-analysis results demonstrated statistical significance with a moderate effect size (SMD = −0.52; 95% CI = −0.75 to −0.30; *p* < 0.0001) and were rated as having low-quality evidence according to GRADE (Supplementary Materials, Table S5). The analysis also showed low heterogeneity (I^2^ = 0%, *p* = 0.56) (Figure 2).

Median duration

The median treatment duration in the included studies was 6 weeks. This duration was used in six studies involving a total of 344 participants (43.22% of the sample). Meta-analysis results indicated statistical significance with a moderate effect size (SMD = −0.72; 95% CI = −1.03 to −0.40; *p* < 0.0001) and were rated as moderate-quality evidence according to GRADE (Supplementary Materials, Table S5). The analysis revealed almost moderate heterogeneity (I^2^ = 42%, *p* = 0.12) (Figure 2).

Maximum duration

This subgroup consisted of studies with treatment durations within 8 to 12 weeks. A total of four studies were included, involving 114 participants (14.32% of the sample). This subgroup demonstrated the large effect size (SMD = −0.88; 95% CI = −1.27 to −0.49; *p* < 0.0001). The evidence quality was rated as moderate according to GRADE (Supplementary Materials, Table S5). Heterogeneity was (I^2^ = 0%, *p* = 0.63) (Figure 2).

Based on the obtained results, we can note that the results increase linearly with the increase in the duration of the treatment, suggesting that the cumulative effects of stabilization have a greater clinical value in extended protocols.

### 3.2. The Influence of Treatment on Type of Pain for Outcome Pain

Specific Low back pain

**Figure 3:**
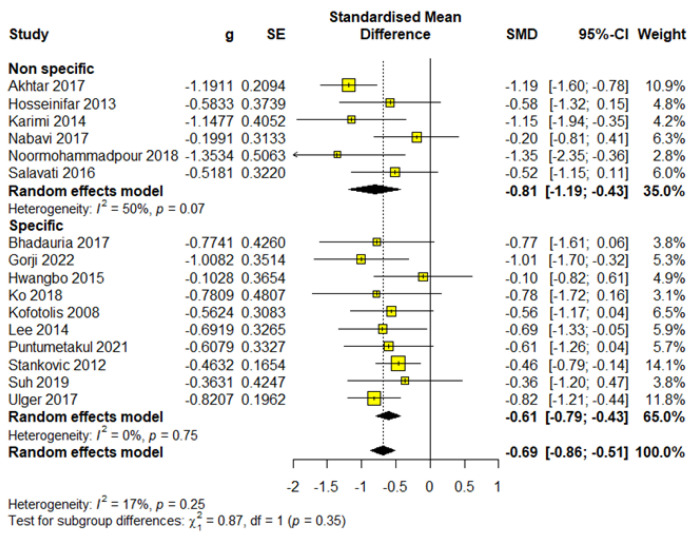
Forest plot for outcome pain—subgroup type of pain.

This subgroup included 528 participants, which accounted for 66.33% of the total sample, distributed across 10 studies. Meta-analysis results demonstrated statistical significance with a moderate effect size (SMD = −0.61; 95% CI = −0.79 to −0.43; *p* < 0.0001) and were rated as having high-quality evidence according to GRADE (Supplementary Materials, Table S5). The analysis showed heterogeneity (I^2^ = 0%, *p* = 0.75) (Figure 3).

Non-specific Low back pain

This subgroup consisted of 268 participants, representing 33.67% of the total sample, across six studies. Meta-analysis results indicated statistical significance with a moderate effect size (SMD = −0.81; 95% CI = −1.19 to −0.43; *p* < 0.0001) and were assessed as high-quality evidence based on the GRADE criteria (Supplementary Materials, Table S5). Additionally, the analysis revealed moderate heterogeneity (I^2^ = 50%, *p* = 0.07) (Figure 3).

Stabilization exercises showed a greater effect in patients with nonspecific pain, which may indicate their greater effectiveness in cases where the etiology of pain is not clearly defined and where biomechanical dysfunction plays a dominant role.

### 3.3. The Impact of Study Quality on Pain

Low risk of bias

Seven studies, comprising 365 participants (45.85% of the total sample), were evaluated as having a low risk of bias. This subgroup demonstrated a large effect size with statistical significance (SMD = −0.89; 95% CI = −1.13 to −0.66; *p* < 0.0001), with evidence quality rated as high according to GRADE (Supplementary Materials, Table S5). The analysis indicated heterogeneity (I^2^ = 1%, *p* = 0.41) (Supplementary Materials, Figure S4).

Moderate risk of bias

Two studies, including 74 participants (9.30% of the total sample), were assessed as having a moderate risk of bias. This subgroup showed a moderate effect size with statistical significance (SMD = −0.57; 95% CI = −1.04 to −0.10; *p* < 0.0001), with the evidence quality rated as low based on GRADE (Supplementary Materials, Table S5). The analysis revealed no heterogeneity (I^2^ = 0%, *p* = 0.97) (Supplementary Materials, Figure S4).

High risk of bias

Seven studies involving 357 participants (44.85% of the total sample) were assessed as having a high risk of bias. This subgroup exhibited a moderate effect size with statistical significance (SMD = −0.51; 95% CI = −0.72 to −0.29; *p* < 0.0001), with the evidence quality classified as very low according to GRADE (Supplementary Materials, Table S5). The analysis showed no heterogeneity (I^2^ = 0%, *p* = 0.48) (Supplementary Materials, Figure S4).

Larger effects reported in studies with low risk of bias confirm the importance of methodological rigor in examining the effects of therapeutic interventions. The higher the risk of bias, the smaller the effects (the opposite of what was expected).

### 3.4. The Influence of Treatment Differences on Pain

Core stability exercises

**Figure 4:**
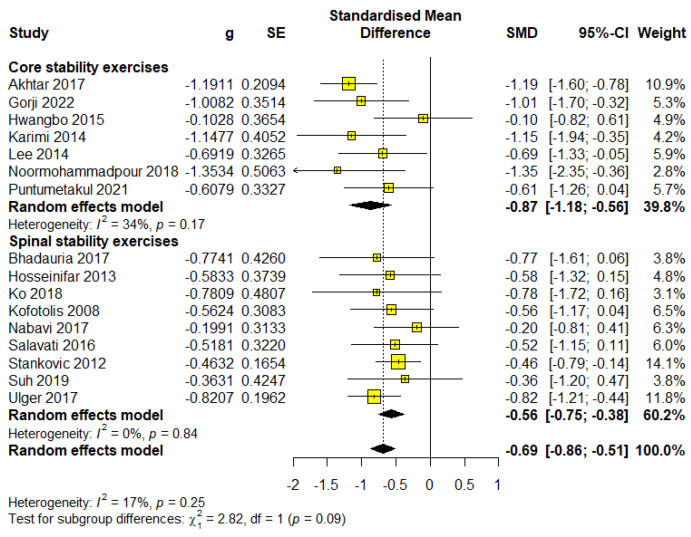
Forest plot for outcome pain—subgroup exercise type.

This subgroup included 262 participants, representing 32.91% of the total sample, across six studies. The meta-analysis results indicated statistical significance with a large effect size (SMD = −0.90; 95% CI = −1.26 to −0.54; *p* < 0.0001) and were classified as moderate-quality evidence based on GRADE (Supplementary Materials, Table S5). The analysis revealed heterogeneity (I^2^ = 42%, *p* = 0.12) (Figure 4).

Spinal stability exercises

This subgroup included 534 participants, representing 67.10% of the total sample, across 10 studies. The meta-analysis results indicated statistical significance with a moderate effect size (SMD = −0.57; 95% CI = −0.75 to −0.40; *p* < 0.0001) and were classified as high-quality evidence based on GRADE (Supplementary Materials, Table S5). The analysis revealed no heterogeneity (I^2^ = 0%, *p* = 0.89) (Figure 4).

Core stability exercises result in greater pain reduction, although spinal stability exercises offer more consistent results with less heterogeneity, making them a more reliable choice in standardized rehabilitation protocols.

### 3.5. The Influence of the Duration of the Treatment on the Disability

Short duration

**Figure 5:**
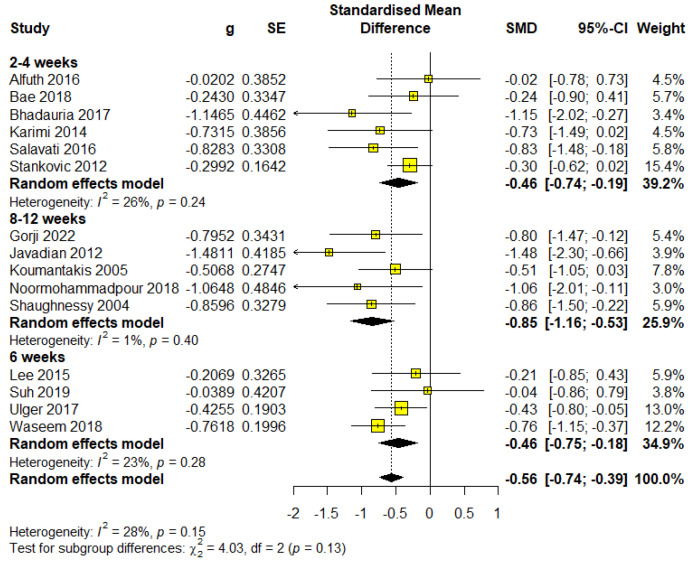
Forest plot for outcome disability—subgroup treatment duration.

The shortest treatment duration was categorized within the 2- to 4-week subgroup. A total of six studies utilized this treatment length, involving 316 participants, which accounted for 40.41% of the total sample. Meta-analysis results demonstrated statistical significance with an almost moderate effect size (SMD = −0.46; 95% CI = −0.74 to −0.19; *p* < 0.0001) and were rated as having low-quality evidence according to GRADE (Supplementary Materials, Table S5). The analysis showed low heterogeneity (I^2^ = 26%, *p* = 0.24) (Figure 5).

Median duration

The median treatment duration in the included studies was 6 weeks. This duration was used in four studies involving 283 participants (36.19% of the sample). Meta-analysis results indicated statistical significance with an almost moderate effect size of the same values by subgroup 2 to 4 weeks (SMD = −0.46; 95% CI = −0.75 to −0.18; *p* < 0.0001) and were rated as moderate-quality evidence according to GRADE (Supplementary Materials, Table S5). The analysis revealed low heterogeneity (I^2^ = 23%, *p* = 0.28) (Figure 5).

Maximum duration

This subgroup consisted of studies with treatment durations within 8 to 12 weeks. A total of five studies were included, involving 183 participants (23.4% of the sample). This subgroup demonstrated the large effect size (SMD = −0.85; 95% CI = −1.16 to −0.53; *p* < 0.0001). The evidence quality was rated high according to GRADE (Supplementary Materials, Table S5). Heterogeneity was I^2^ = 1%, *p* = 0.40 (Figure 5).

The effect on disability also increases with the length of treatment, indicating the importance of continuity and progression of exercises in improving the functional status of patients.

### 3.6. The Influence of Treatment on Type of Pain for Outcome Disability

Specific Low back pain

**Figure 6:**
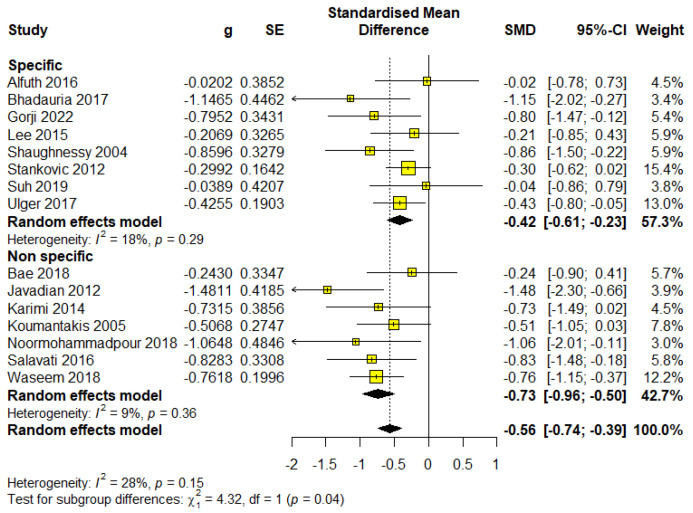
Forest plot for outcome disability—subgroup type of pain.

This subgroup included 464 participants, which accounted for 59.34% of the total sample, distributed across eight studies. Meta-analysis results demonstrated statistical significance with an almost moderate effect size (SMD = −0.42; 95% CI = −0.61 to −0.23; *p* < 0.0001) and were rated as having moderate-quality evidence according to GRADE (Supplementary Materials, Table S5). The analysis showed heterogeneity (I^2^ = 18%, *p* = 0.29) (Figure 6).

Non-specific Low back pain

This subgroup included 318 participants, representing 40.66% of the total sample, across seven studies. Meta-analysis results indicated statistical significance with an almost large effect size (SMD = −0.73; 95% CI = −0.96 to −0.50; *p* < 0.0001) and were assessed as moderate-quality evidence based on the GRADE criteria (Supplementary Materials, Table S5). Additionally, the analysis revealed very low heterogeneity (I^2^ = 9%, *p* = 0.36) (Figure 6).

Patients with non-specific pain experience a greater reduction in disability, which confirms their high responsiveness to targeted stabilization exercises.

### 3.7. The Impact of Study Quality on Disability

Low risk of bias

Eight studies, comprising 420 participants (53.70% of the total sample), were evaluated as having a low risk of bias. This subgroup demonstrated a moderate size with statistical significance (SMD = −0.63; 95% CI = −0.83 to −0.44; *p* < 0.0001), with evidence quality rated as high according to GRADE (Supplementary Materials, Table S5). The analysis did not show heterogeneity (I^2^ = 0%, *p* = 0.48) (Supplementary Materials, Figure S8).

High risk of bias

Seven studies involving 362 participants (46.29% of the total sample) were assessed as having a high risk of bias. This subgroup exhibited a moderate effect size with statistical significance (SMD = −0.50; 95% CI = −0.81 to −0.18; *p* < 0.0001), with the evidence quality classified as very low according to GRADE (Supplementary Materials, Table S5). The analysis showed moderate heterogeneity (I^2^ = 46%, *p* = 0.08) (Supplementary Materials, Figure S8).

Studies with higher methodological quality consistently show a more pronounced effect on disability reduction, emphasizing the importance of controlling for bias factors.

### 3.8. The Impact of Treatment Differences on Disability

Core stability exercises

**Figure 7:**
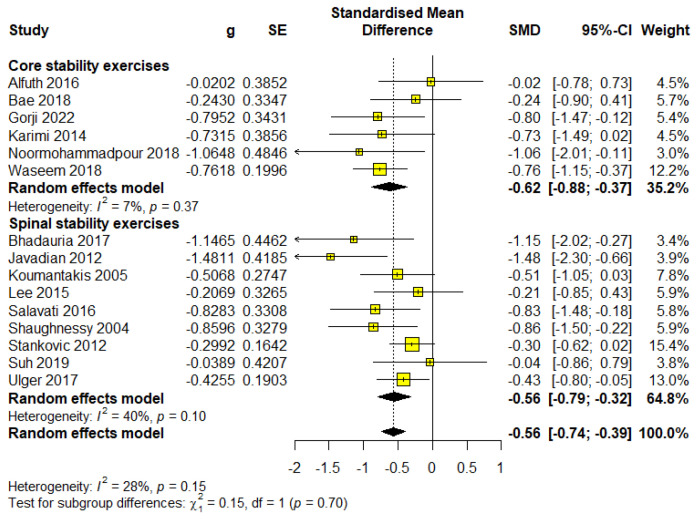
Forest plot for outcome disability—subgroup exercise type.

This subgroup consisted of 257 participants, representing 34.18% of the total sample, across six studies. The meta-analysis results indicated statistical significance with a moderate effect size (SMD = −0.62; 95% CI = −0.88 to −0.37; *p* < 0.0001) and were classified as low-quality evidence based on GRADE (Supplementary Materials, Table S5). The analysis revealed negligible heterogeneity (I^2^ = 7%, *p* = 0.37) (Figure 7).

Spinal stability exercises

This subgroup consisted of 525 participants, representing 62.06% of the total sample, across nine studies. The meta-analysis results indicated statistical significance with an almost moderate effect size (SMD = −0.56; 95% CI = −0.79 to −0.32; *p* < 0.0001) and were classified as high-quality evidence based on GRADE (Supplementary Materials, Table S5). The analysis revealed negligible heterogeneity (I^2^ = 9%, *p* = 0.36) (Figure 7).

Although both approaches are effective, core stability exercises have a slightly stronger effect, while spinal stability shows more stable results with high-quality evidence.

## 4. Discussion

In our meta-analysis, we pooled the results of 23 studies to obtain the effect size of stabilization exercise for treating CLBP in subjects in reducing pain and disability. The effect size for the outcome pain ranged from 0.51 to 0.89, depending on the analysis, while for disability it ranged from 0.42 to 0.85, which clearly shows that stabilization exercises have positive effects in reducing pain and disability. Sensitivity analysis shows that the effect for outcome pain, pooled results, ranged from 0.62 to 0.72, the heterogeneity varied from 0% to 23%, and for the outcome disability from 0.52 to 061 the heterogeneity varied from 9% to 33% (Supplementary Materials). The meta-analysis revealed that longer treatment durations (8–12 weeks) showed the strongest effects on pain reduction (SMD = −0.88) and disability improvement (SMD = −0.85). For pain type, non-specific low back pain responded better (SMD = −0.81 for pain, −0.73 for disability) compared to specific LBP (SMD = −0.61 and −0.42, respectively). The 6-week duration also demonstrated moderate effects (SMD = −0.72 for pain). Core stability exercises had superior pain reduction (SMD = −0.90, large effect) compared to spinal stability exercises (SMD = −0.57), while spinal stability exercises showed higher-quality evidence for disability improvement (SMD = −0.56, high-quality) versus core stability (SMD = −0.62, low-quality).

The results of this meta-analysis suggest that the duration of stabilization exercise interventions significantly influences pain reduction in patients with CLBP. The shortest duration demonstrated a moderate effect size with low-quality evidence. The moderate effect despite low-quality evidence may reflect the immediate but limited impact of short-term interventions, emphasizing the need for more rigorous studies in this subgroup. In contrast, the median duration showed a greater effect size with moderate-quality evidence, suggesting that extending the intervention enhances its effectiveness. The maximum duration exhibited the largest effect size with moderate-quality evidence, indicating that longer interventions might optimize pain relief. Similar trends were observed for disability outcomes. Shorter durations resulted in an almost moderate effect size with low-quality evidence. The median duration maintained the same effect size but with moderate-quality evidence, suggesting more reliable benefits for disability reduction. The maximum duration demonstrated a large effect size with high-quality evidence, emphasizing the substantial impact of prolonged interventions. The meta-analysis revealed that stabilization exercises significantly reduced pain, both specific and non-specific low back pain. For specific low back pain, a moderate effect size was observed with high-quality evidence, while non-specific pain demonstrated a large effect size, also with high-quality evidence. Results revealed that stabilization exercises significantly reduced pain and disability in both specific and non-specific low back pain. For specific low back pain, an almost moderate effect size was observed with moderate-quality evidence, while non-specific pain demonstrated an almost large effect size with moderate-quality evidence. These findings suggest that stabilization exercises may be more effective for reducing pain and disability in non-specific low back pain, potentially due to their comprehensive impact on spinal stability and functional capacity. Core stability exercises exhibited a large effect size for pain with moderate-quality evidence but a moderate effect size for disability with low-quality evidence. Spinal stability exercises showed a moderate effect size for pain and an almost moderate effect for disability, both supported by high-quality evidence. In both cases, core stability exercises proved to be better. Subgroups with a low risk of bias reported the large effect size for pain with high-quality evidence and the moderate effect size for disability with moderate-quality evidence, suggesting that well-conducted studies show more substantial benefits. Conversely, studies with a high risk of bias demonstrated moderate effect sizes with very low to moderate-quality evidence, indicating that methodological limitations likely diluted the observed effects. Clinicians should prioritize 8–12 week interventions for chronic low back pain, as they yielded the largest improvements in both pain relief (SMD = −0.88) and disability reduction (SMD = −0.85). Shorter programs (2–4 weeks) may offer modest benefits but are less effective for long-term outcomes. For time-constrained settings, a 6-week program (SMD = −0.72 for pain) balances efficacy and feasibility, though longer durations are ideal where possible. Core stability exercises are particularly effective for pain reduction (SMD = −0.90) and may be favored for patients with pain. Spinal stability exercises are supported by higher-quality evidence for improving disability (SMD = −0.56) and are recommended for functional rehabilitation, especially in non-specific CLBP. Patients with non-specific CLBP showed stronger responses to treatment (SMD = −0.81 for pain, −0.73 for disability) than those with specific LBP. While core stability had larger effect sizes for pain, spinal stability’s higher GRADE ratings (high-quality evidence) support its use in standardized protocols. Clinicians should weigh rapid pain relief (core) against functional recovery (spinal) based on patient goals.

Our answer to the posed PICOS question would be as follows: In patients with CLBP, stabilization exercise therapy demonstrates clinically significant reductions in pain and improvements in functional status compared to other treatments, with optimal outcomes achieved through 8–12 week interventions, as evidenced by randomized controlled trials.

### 4.1. Comparison with Other Meta-Analyses and Treatments

The study by [65] on manipulation and mobilization showed a small effect on pain relief (SMD = 0.28) and disability reduction (SMD = 0.33) in chronic low back pain. The effects were consistent but smaller compared to stabilization exercises in our study. In the study by [66], osteopathic manipulative treatment showed a small effect on disability reduction (SMD = 0.36) in nonspecific low back pain. The effects were smaller than those observed in our study. In the study by [67], dry needling showed limited effectiveness for low back pain, with effects notably smaller than those of stabilization exercises in our analysis. In the study by [68], home exercise training showed a large effect on pain relief in nonspecific low back pain and almost as large for disability; however, in both analyses the heterogeneity was 90.2%, and these results cannot be relevant. The study by [69], strength exercise interventions, showed moderate effects on pain relief (SMD = 0.50), while combined exercises did not affect pain reduction (SMD = 0.16), while the overall application of different exercises had small effects (SMD = 0.32) in chronic low back pain. The effects were smaller than those observed in our study. In a study by [70], the myofascial release technique has no effect in reducing pain (SMD = 0.12), while small effects appear in reducing disability (SMD = 0.35). In this case, stabilization exercises are also shown to be a better choice in treating pain and disability. Meta-analyses [71,72,73] use mean difference, so the results cannot be compared with ours.

### 4.2. Clinical Recommendations and Implications

Based on the GRADE assessment, the following clinical recommendations and implications can be drawn: Stabilization exercises are strongly recommended for both specific and non-specific CLBP, as they provide significant pain relief and functional improvement. Clinicians should prioritize interventions supported by high-quality evidence, as they are more reliable and effective. These exercises are more effective than manipulation/mobilization, osteopathic treatment, home-based exercises, strength exercises, and myofascial release. While stabilization exercises are highly effective, they can be combined with other therapies (e.g., manual therapy, education, or psychological support) for a multimodal approach, especially in complex cases. Interventions with small or inconsistent effects (e.g., manipulation/mobilization, osteopathic treatment, myofascial release) should be used as adjuncts rather than primary treatments, particularly when stabilization exercises are available. Longer-duration programs (8–12 weeks) are highly effective and should be implemented for sustained pain relief and disability reduction. Core stability exercises are effective for pain relief and should be included in rehabilitation programs, though further research is needed to strengthen the evidence for disability reduction. Short-duration programs (2–4 weeks) may provide initial benefits but are insufficient for long-term outcomes. They should be supplemented with longer-duration interventions. Moderate risk of bias study findings from these studies should be interpreted with caution due to lower evidence quality. Studies with a high risk of bias yield less reliable results and should be used with caution in clinical decision-making.

### 4.3. Strengths and Limitations

Our study included a wide range of subgroups (e.g., treatment duration, type of pain, study quality, and exercise type), providing a detailed and nuanced understanding of the effectiveness of stabilization exercises. Many of our findings were supported by moderate- to high-quality evidence according to the GRADE tool, particularly for specific and non-specific low back pain, longer-duration programs, and low-risk-of-bias studies. This strengthens the reliability of our conclusions. Our study focused on both pain relief and disability reduction, which are critical outcomes for patients with CLBP and are highly relevant to clinical practice.

The effect sizes for pain relief (SMD = −0.52 to −0.88) and disability reduction (SMD = −0.46 to −0.85) were moderate to large, indicating that stabilization exercises are highly effective interventions for CLBP. Many subgroups showed low heterogeneity, suggesting consistent results across studies and increasing confidence in the findings. Our study provides clear clinical recommendations, such as prioritizing longer-duration (8–12 weeks) and supervised stabilization exercises, which can directly inform treatment protocols.

While some subgroups had high-quality evidence, others (e.g., short-duration programs, high-risk-of-bias studies) were supported by low- or very low-quality evidence, limiting the reliability of findings in these areas. Certain subgroups (e.g., non-specific LBP for pain outcomes, I^2^ = 50%) showed moderate heterogeneity, indicating variability in study designs, interventions, or populations that could affect the consistency of results. Most studies focused on short- to medium-term outcomes (up to 12 weeks). There is a lack of data on the long-term effectiveness of stabilization exercises beyond 12 weeks. The included studies used varying protocols for stabilization exercises (e.g., core stability vs. spinal stability exercises), which may introduce variability in the results and make it difficult to identify the most effective approach. The findings may not be generalizable to all populations, as the included studies may have excluded certain groups (e.g., elderly patients, those with severe comorbidities, or specific subtypes of LBP). The meta-analysis relied on aggregate data from studies rather than individual patient data, which limits the ability to perform more detailed subgroup analyses (e.g., by age, gender, or baseline pain severity). Several subgroups had a small number of respondents (below 400), which contributed to imprecision in the assessment of the strength of evidence.

## 5. Conclusions

This meta-analysis demonstrates that stabilization exercises are a highly effective intervention for managing chronic low back pain, offering moderate to large effect sizes for both pain relief (SMD = −0.52 to −0.88) and disability reduction (SMD = −0.46 to −0.85). Compared to other interventions such as manipulation/mobilization, osteopathic manipulative treatment, home exercise training, strength exercises, and myofascial release, stabilization exercises consistently show superior results. Longer-duration programs (8–12 weeks) and supervised, structured protocols yield the best results, making them a first-line treatment for CLBP. The high-quality evidence supporting stabilization exercises, particularly for specific and non-specific low back pain, underscores their reliability and clinical relevance. In contrast, other interventions, such as home exercise training, show high heterogeneity, making their results less reliable, while interventions like manipulation/mobilization and myofascial release demonstrate smaller effects and are better suited as adjunct therapies. These findings highlight the importance of prioritizing active, supervised, and longer-duration stabilization programs in clinical practice. Future research should focus on addressing heterogeneity in other interventions, exploring long-term outcomes, and standardizing protocols to further strengthen the evidence base. Overall, stabilization exercises represent the most effective and evidence-based approach for improving pain and function in patients with chronic low back pain.

## Figures and Tables

**Table 1:** PICOS Eligibility Inclusion/Exclusion Criteria.

**Category**	**Inclusion Criteria**
Population	Adults aged 18–65 years with CLBP
Intervention	Core stabilization exercises, lumbar stabilization exercises,
	trunk stabilization exercises, or spinal stabilization exercises
Comparator	Control group (no treatment, other exercise treatments,
	or home exercises)
Outcomes	Pain and disability
Study Design	Randomized controlled trials
Other	No language restrictions. Date of publication: studies that
	were published after 2000.
**Reason**	**Exclusion Criteria**
1	Trials with the same exercise intervention type with minor
	differences in the application protocol.
2	Studies without a control group as a comparator.
3	Systematic reviews, meta-analyses, study protocols, books,
	book reviews, and conference publications.
4	Studies that used another type of therapy.
5	Acute or subacute low back pain.

## Data Availability

All data were obtained from published studies. Additional details are available from the corresponding author upon request.

## Supplementary Materials

The following supporting information can be downloaded at: https://www.mdpi.com/article/10.3390/healthcare13090960/s1, File S1 (PDF), including the PRISMA 2020 Checklist, Search Strategy (Tables S1–S4), Supplementary Results, Figures, and GRADE Assessments.

## Abbreviations

The following abbreviations are used in this manuscript:

CLBP	Chronic Low Back Pain
LBP	Low Back Pain
RCT	Randomized Controlled Trial
PRISMA	Preferred Reporting Items for Systematic Reviews and Meta-Analyses
GRADE	Grading of Recommendations, Assessment, Development, and Evaluation
SMD	Standardized Mean Difference
CI	Confidence Interval
ODQ	Oswestry Disability Questionnaire
RM	Roland–Morris Disability Questionnaire
VAS	Visual Analogue Scale
NRS	Numerical Rating Scale
VRS	Verbal Rating Scale
PICOS	Population, Intervention, Comparator, Outcomes, Study Design
IÂ2	Higgins’ I-squared (statistical heterogeneity)
PROSPERO	International Prospective Register of Systematic Reviews
MDPI	Multidisciplinary Digital Publishing Institute
R	Statistical software environment
