# Supplementary material for: Pain and Disability Therapy with Stabilization Exercises in Patients with Chronic Low Back Pain: A Meta-Analysis
Source: Healthcare (Basel). 2025 Apr 22;13(9):960. doi: 10.3390/healthcare13090960 (PMC12072060; doi:10.3390/healthcare13090960)
Supplement: Supplementary file 1 [file healthcare-13-00960-s001.zip › healthcare-3582167-supplementary.pdf]

Contents

1. PRISMA 2020 Checklist 2
2. Search strategy 4
Table 1: Pubmed 4
Table 2: Web of Science 4
Table 3: Scopus 4
Table 4: Cochrane library 4
3. Supplementary results 5
4. Figures 6
5. GRADE assessments 12

1. PRISMA 2020 Checklist

Section and Topic	Item #	Checklist item	Location where item is reported
**TITLE**			
Title	1	Identify the report as a systematic review.	1
**ABSTRACT**			
Abstract	2	See the PRISMA 2020 for Abstracts checklist.	1
**INTRODUCTION**			
Rationale	3	Describe the rationale for the review in the context of existing knowledge.	2
Objectives	4	Provide an explicit statement of the objective(s) or question(s) the review addresses.	2
**METHODS**			
Eligibility criteria	5	Specify the inclusion and exclusion criteria for the review and how studies were grouped for the syntheses.	3
Information sources	6	Specify all databases, registers, websites, organisations, reference lists and other sources searched or consulted to identify studies. Specify the date when each source was last searched or consulted.	3
Search strategy	7	Present the full search strategies for all databases, registers and websites, including any filters and limits used.	3-4
Selection process	8	Specify the methods used to decide whether a study met the inclusion criteria of the review, including how many reviewers screened each record and each report retrieved, whether they worked independently, and if applicable, details of automation tools used in the process.	3-4
Data collection process	9	Specify the methods used to collect data from reports, including how many reviewers collected data from each report, whether they worked independently, any processes for obtaining or confirming data from study investigators, and if applicable, details of automation tools used in the process.	3-4
Data items	10a	List and define all outcomes for which data were sought. Specify whether all results that were compatible with each outcome domain in each study were sought (e.g. for all measures, time points, analyses), and if not, the methods used to decide which results to collect.	3-4
	10b	List and define all other variables for which data were sought (e.g. participant and intervention characteristics, funding sources). Describe any assumptions made about any missing or unclear information.	
Study risk of bias assessment	11	Specify the methods used to assess risk of bias in the included studies, including details of the tool(s) used, how many reviewers assessed each study and whether they worked independently, and if applicable, details of automation tools used in the process.	3-4
Effect measures	12	Specify for each outcome the effect measure(s) (e.g. risk ratio, mean difference) used in the synthesis or presentation of results.	3-4
Synthesis methods	13a	Describe the processes used to decide which studies were eligible for each synthesis (e.g. tabulating the study intervention characteristics and comparing against the planned groups for each synthesis (item #5)).	
	13b	Describe any methods required to prepare the data for presentation or synthesis, such as handling of missing summary statistics, or data conversions.	3-4
	13c	Describe any methods used to tabulate or visually display results of individual studies and syntheses.	3-4
	13d	Describe any methods used to synthesize results and provide a rationale for the choice(s). If meta-analysis was performed, describe the model(s), method(s) to identify the presence and extent of statistical heterogeneity, and software package(s) used.	3-4
	13e	Describe any methods used to explore possible causes of heterogeneity among study results (e.g. subgroup analysis, meta-regression).	3-4
	13f	Describe any sensitivity analyses conducted to assess robustness of the synthesized results.	3-4
Reporting bias assessment	14	Describe any methods used to assess risk of bias due to missing results in a synthesis (arising from reporting biases).	3-4
Certainty assessment	15	Describe any methods used to assess certainty (or confidence) in the body of evidence for an outcome.	3-4

Section and Topic	Item #	Checklist item	Location where item is reported
**RESULTS**			
Study selection	16a	Describe the results of the search and selection process, from the number of records identified in the search to the number of studies included in the review, ideally using a flow diagram.	5
	16b	Cite studies that might appear to meet the inclusion criteria, but which were excluded, and explain why they were excluded.	5
Study characteristics	17	Cite each included study and present its characteristics.	8-9
Risk of bias in studies	18	Present assessments of risk of bias for each included study.	7
Results of individual studies	19	For all outcomes, present, for each study: (a) summary statistics for each group (where appropriate) and (b) an effect estimate and its precision (e.g. confidence/credible interval), ideally using structured tables or plots.	10-15
Results of syntheses	20a	For each synthesis, briefly summarise the characteristics and risk of bias among contributing studies.	6
	20b	Present results of all statistical syntheses conducted. If meta-analysis was done, present for each the summary estimate and its precision (e.g. confidence/credible interval) and measures of statistical heterogeneity. If comparing groups, describe the direction of the effect.	10-15
	20c	Present results of all investigations of possible causes of heterogeneity among study results.	10-15
	20d	Present results of all sensitivity analyses conducted to assess the robustness of the synthesized results.	14
Reporting biases	21	Present assessments of risk of bias due to missing results (arising from reporting biases) for each synthesis assessed.	14
Certainty of evidence	22	Present assessments of certainty (or confidence) in the body of evidence for each outcome assessed.	
**DISCUSSION**			
Discussion	23a	Provide a general interpretation of the results in the context of other evidence.	16
	23b	Discuss any limitations of the evidence included in the review.	16-17
	23c	Discuss any limitations of the review processes used.	16-17
	23d	Discuss implications of the results for practice, policy, and future research.	17
**OTHER INFORMATION**			
Registration and protocol	24a	Provide registration information for the review, including register name and registration number, or state that the review was not registered.	CRD42024502701
	24b	Indicate where the review protocol can be accessed, or state that a protocol was not prepared.	
	24c	Describe and explain any amendments to information provided at registration or in the protocol.	
Support	25	Describe sources of financial or non-financial support for the review, and the role of the funders or sponsors in the review.	18
Competing interests	26	Declare any competing interests of review authors.	18
Availability of data, code and other materials	27	Report which of the following are publicly available and where they can be found: template data collection forms; data extracted from included studies; data used for all analyses; analytic code; any other materials used in the review.	data extract form included studies

2. Search strategy

Table 1: Pubmed

Database	Query	Filters
PubMed	("low back pain"[MeSH Terms] OR "low back pain"[Title/Abstract] OR "back pain"[MeSH Terms] OR "back pain"[Title/Abstract] OR "LBP"[Title/Abstract] OR "lumbago"[Title/Abstract] OR "backache"[Title/Abstract]) AND ("conservative treatment"[MeSH Terms] OR "conservative treatment"[Title/Abstract] OR "exercise therapy"[MeSH Terms] OR "exercise therapy"[Title/Abstract] OR "stabilization exercise"[Title/Abstract] OR "core stabilization"[Title/Abstract] OR "physical exercise"[Title/Abstract] OR "lumbar stabilization"[Title/Abstract] OR "trunk stabilization"[Title/Abstract] OR "spinal stabilization"[Title/Abstract]) AND ("randomized controlled trial"[Publication Type] OR "RCT"[Title/Abstract])	Publication Date: 2000–2025<br>Article Type: Randomized Controlled Trials<br>Language: English, German,

Table 2: Web of Science

Database	Query	Filters
Web of Science	(TS=(low back pain)) OR TS=(back pain)) OR TS=(LBP)) OR TS=(lumbago)) OR TS=(lumb pain)) OR TS=(backache)) AND TS=(conservative treatment)) OR TS=(exercise therapy)) OR TS=(stabilization exercise)) OR TS=(core stabilization)) OR TS=(physical exercise)) OR TS=(lumbar stabilization)) OR TS=(trunk stabilization)) OR TS=(spinal stabilization)) AND TS=(randomized controlled trial)) OR TS=(RCT)	Publication years: 2000–2025<br>Document Type: Article,<br>Research Areas: Rehabilitation, Orthopedics, Sports Science<br>Language: English, Spanish, German, Portugese

Table 3: Scopus

Database	Query	Filters
Scopus	Article title, Abstract, Keywords (("low back pain" OR "back pain" OR "LBP" OR "lumbago" OR "lumb pain" OR "backache") AND ("conservative treatment" OR "exercise therapy" OR "stabilization exercise" OR "core stabilization" OR "physical exercise" OR "lumbar stabilization" OR "trunk stabilization" OR "spinal stabilization") AND ("randomized controlled trial" OR "RCT"))	Date Range: 2000–2025,<br>Document Type: Article<br>Subject Areas: Medicine, Health professions<br>Language: English, Spanish, German, French

Table 4: Cochrane Library

Database	Query	Filters
Cochrane Library	("low back pain" OR "back pain" OR "LBP" OR "lumbago" OR "lumb pain" OR "backache") AND ("conservative treatment" OR "exercise therapy" OR "stabilization exercise" OR "core stabilization" OR "physical exercise" OR "lumbar stabilization" OR "trunk stabilization" OR "spinal stabilization") AND ("randomized controlled trial" OR "RCT")	Trials: Include randomized controlled trials, Date Range: 2000–2025<br>Language: English

3. Supplementary results

The overall effect of stabilization exercise for outcome pain

The number of studies that measured the outcome of pain was 16. There were a total of 796 subjects, of which 419 (52.64%) were in the experimental group and 377 (47.36%) in the control group. The performed analysis shows statistically significant results with a moderate effect size (SMD = -0.69; 95% CI = -0.86, -0.51; $p < 0.0001$; GRADE: moderate (Supplementary materials, Table 5) and heterogeneity ($I^2 = 17\%$, $p = 0.25$) (Supplementary materials, Figure 3). Sensitivity analysis shows that the effect ranged from 0.62 to 0.72, and the heterogeneity varied from 0% to 23% (Supplementary materials, Figure 5). Publication bias was not statistically significant by Egger's test (intercept -0.06; 95% CI = -1.76, -1.64; $p = 0.95$) (Supplementary materials, Figure 6).

The overall effect of stabilization exercise for outcome disability

The number of studies that measured the outcome of disability was 15. There were a total of 781 subjects, of which 415 (53.14%) were in the experimental group and 367 (46.99%) were in the control group. The performed analysis shows statistically significant results with a moderate effect size (SMD = -0.56; 95% CI = -0.74, -0.39; $p < 0.0001$; GRADE: moderate (Supplementary materials, Table 5) and heterogeneity ($I^2 = 28\%$, p = 0.15) (Supplementary materials, Figure 7). Sensitivity analysis shows that the effect ranged from 0.52 to 0.61, and the heterogeneity varied from 9% to 33% (Supplementary materials, Figure 9). Publication bias was not statistically significant by Egger's test (intercept -1.24; 95% CI = -2.97, -0.48; $p = 0.18$) (Supplementary materials, Figure 10).

4. Figures

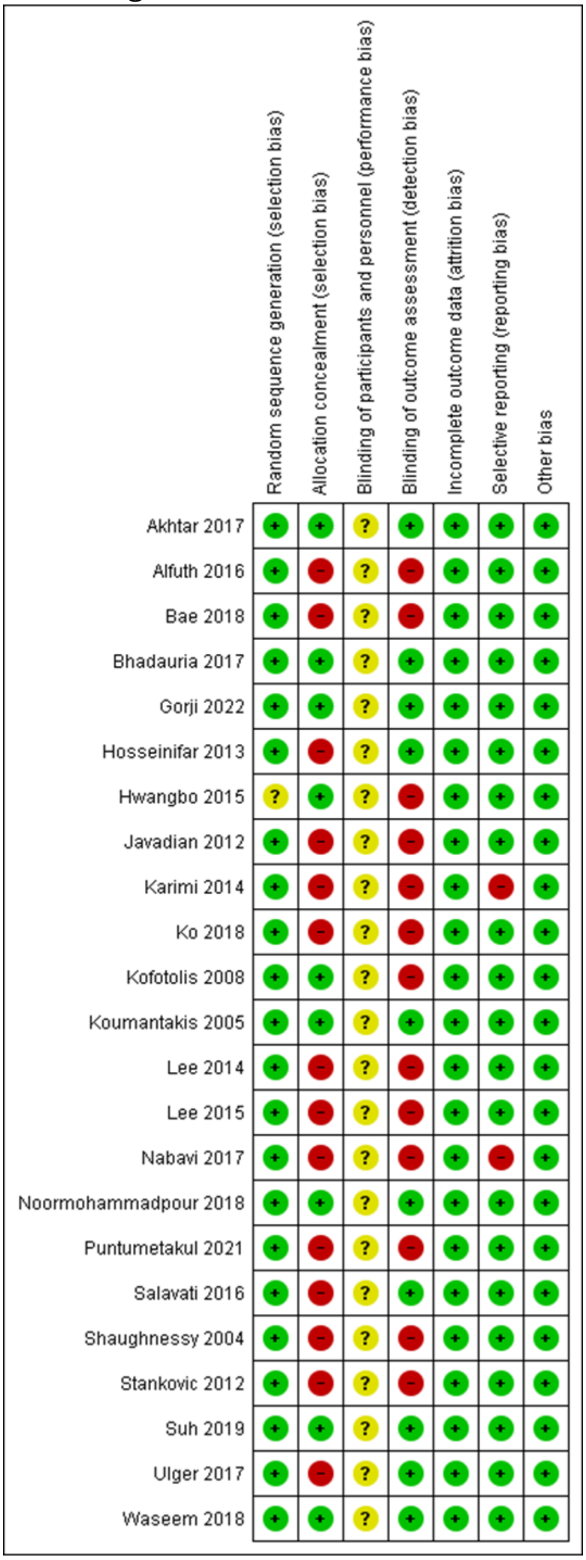

Figure 1. Risk of bias for randomization studies: 1. Random sequence generation (selection bias); 2. Allocation concealment (selection bias); 3. Blinding of participants and personnel (performance bias); 4. Blinding of outcome assessment (detection bias); 5. Incomplete outcome data (attrition bias); 6. Selective reporting (reporting bias); 7. Other bias. Green is low risk, yellow is unclear risk, and red is high risk.

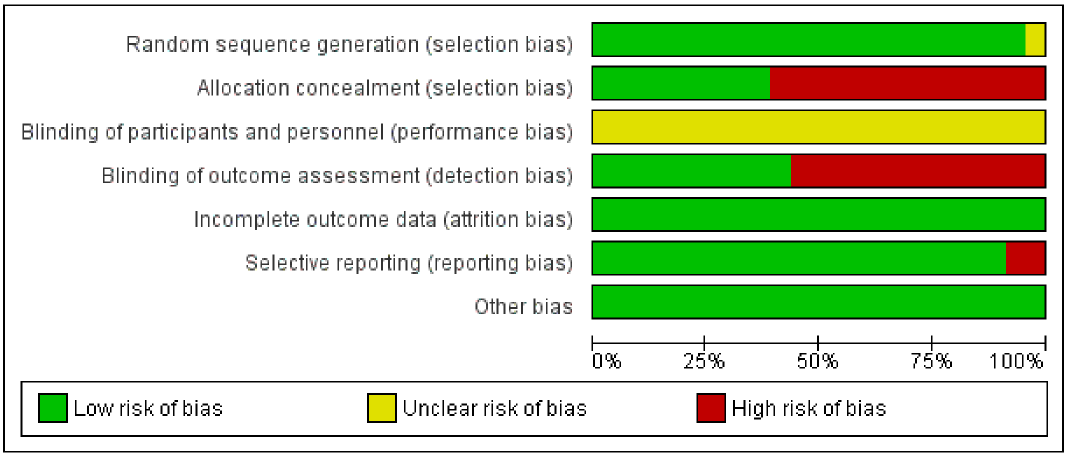

Figure 2. Risk of bias graph: review authors' judgments about each risk of bias item presented as percentages across all included studies

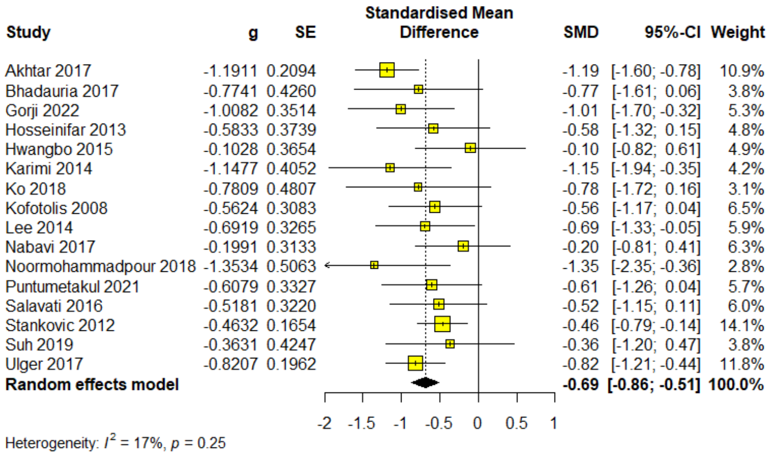

Figure 3. Forest plot for outcome Pain – Pooled

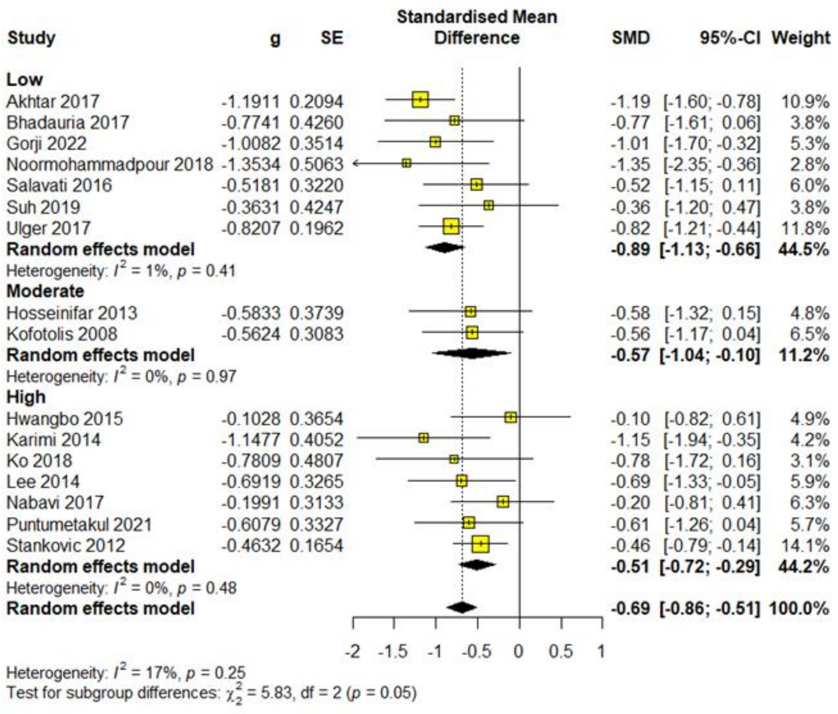

Figure 4. Forest plot for outcome Pain – Subgroup risk of bias

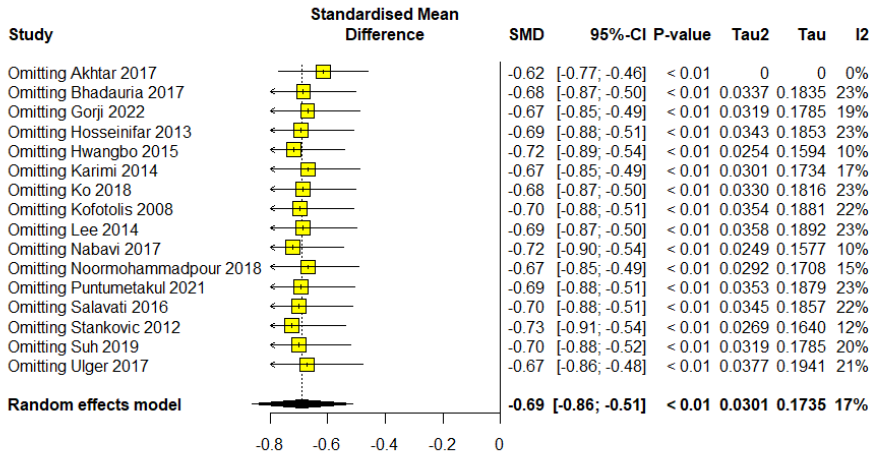

Figure 5. Leave-one-out meta-analysis for outcome Pain

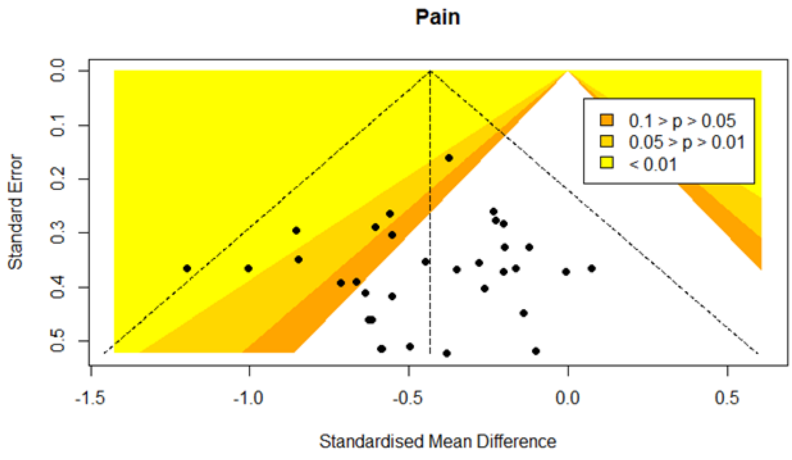

Figure 6. Funnel plot for outcome Pain

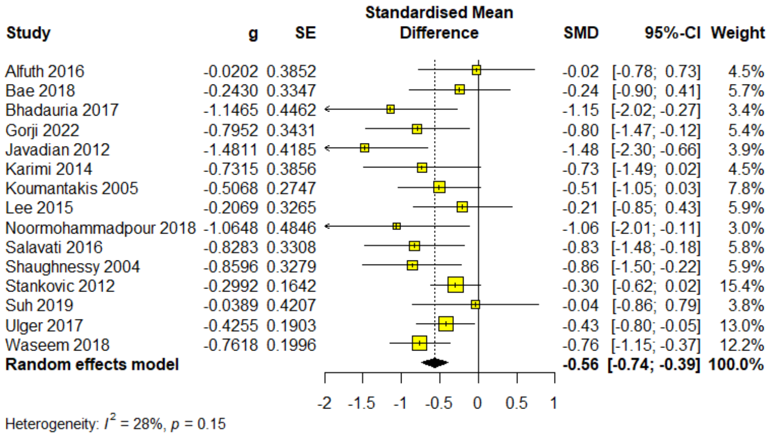

Figure 7. Forest plot for outcome Disability – Pooled

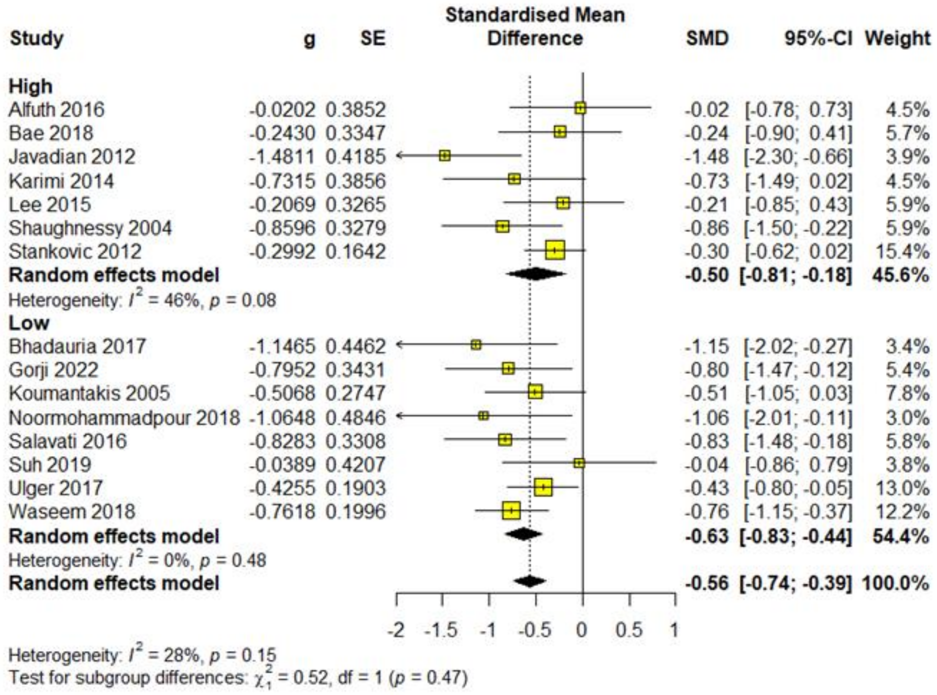

Figure 8. Forest plot for outcome Disability – Subgroup risk of bias

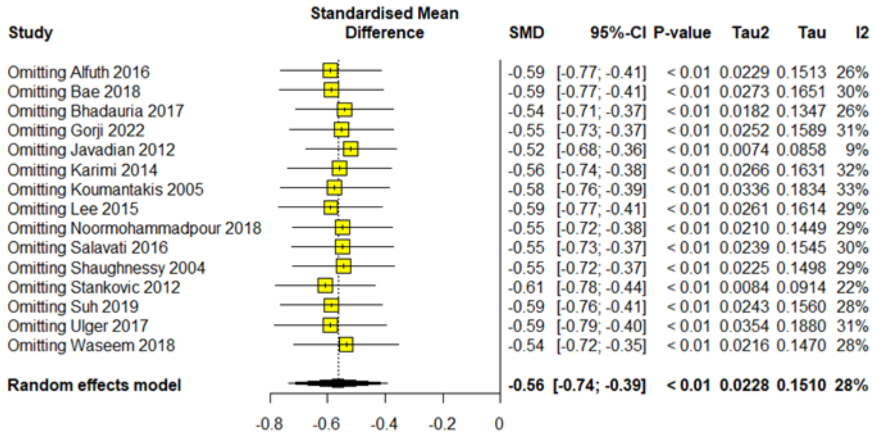

Figure 9. Leave-one-out meta-analysis for outcome Disability

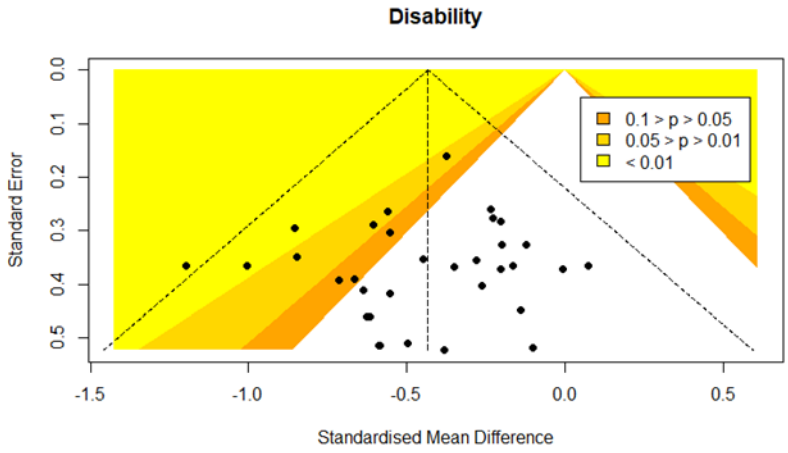

Figure 10. Funnel plot for outcome Disability.

5. GRADE assessments

Table 5: GRADE assessments

Certainty assessment							№ of patients		Effect		Certainty	Importance
**№ of studies**	**Study design**	**Risk of bias**	**Inconsistency**	**Indirectness**	**Imprecision**	**Other considerations**	**[Intervention]**	**[Comparison]**	**Relative (95% CI)**	**Absolute (95% CI)**		
**All stabilization exercises (Pain) (follow-up: range 2 weeks to 12 weeks)**												
16	Randomised trials	serious[a]	not serious	not serious	not serious	none	419	377	-	SMD **0.69 SD lower** (0.86 lower to 0.51 lower)	⊕⊕⊕◯ **Moderate**[a]	CRITICAL
**Short duration (Pain) (follow-up: range 2 weeks to 4 weeks)**												
6	Randomised trials	serious[b]	not serious	not serious	serious[c]	none	191	147	-	SMD **0.52 SD lower** (0.75 lower to 0.3 lower)	⊕⊕◯◯ **Low**[b,c]	CRITICAL
**Median duration (Pain) (follow-up: median 6 weeks)**												
6	Randomised trials	not serious	not serious	not serious	serious[d]	none	170	174	-	SMD **0.72 SD lower** (1.03 lower to 0.4 lower)	⊕⊕⊕◯ **Moderate**[d]	CRITICAL
**Maximum duration (Pain) (follow-up: range 8 weeks to 12 weeks)**												
4	Randomised trials	serious[e]	not serious	not serious	serious[f]	strong association	58	56	-	SMD **0.88 SD lower** (1.27 lower to 0.49 lower)	⊕⊕⊕◯ **Moderate**[e,f]	CRITICAL
**Specific LBP (Pain)**												
10	Randomised trials	not serious	not serious	not serious	not serious	none	285	243	-	SMD **0.61 SD lower** (0.79 lower to 0.43 lower)	⊕⊕⊕⊕ **High**	CRITICAL
**Non specific LBP (Pain)**												

Certainty assessment							№ of patients		Effect		Certainty	Importance
**№ of studies**	**Study design**	**Risk of bias**	**Inconsistency**	**Indirectness**	**Imprecision**	**Other considerations**	**[Intervention]**	**[Comparison]**	**Relative (95% CI)**	**Absolute (95% CI)**		
6	Randomised trials	not serious	not serious	not serious	serious[g]	strong association	134	134	-	SMD **0.81 SD lower** (1.19 lower to 0.43 lower)	⊕⊕⊕⊕ **High[g]**	CRITICAL
**Core stability exercises (Pain)**												
6	Randomised trials	serious[h]	not serious	not serious	serious[i]	strong association	131	131	-	SMD **0.9 SD lower** (1.26 lower to 0.54 lower)	⊕⊕⊕◯ **Moderate[h,i]**	CRITICAL
**Spinal stability exercises (Pain)**												
10	Randomised trials	not serious	not serious	not serious	not serious	none	288	246	-	SMD **0.57 SD lower** (0.75 lower to 0.4 lower)	⊕⊕⊕⊕ **High**	CRITICAL
**Low risk (Pain)**												
7	Randomised trials	not serious	not serious	not serious	serious[j]	strong association	181	184	-	SMD **0.89 SD lower** (1.13 lower to 0.66 lower)	⊕⊕⊕⊕ **High[j]**	CRITICAL
**Moderate risk (Pain)**												
2	Randomised trials	not serious	not serious	not serious	very serious[k]	none	38	36	-	SMD **0.57 SD lower** (1.04 lower to 0.1 lower)	⊕⊕◯◯ **Low[k]**	IMPORTANT
**High risk (Pain)**												
7	Randomised trials	very serious[l]	not serious	not serious	serious[m]	none	200	157	-	SMD **0.51 SD lower** (0.72 lower to 0.29 lower)	⊕◯◯◯ **Very low[l,m]**	IMPORTANT
**All stabilization exercises (Disability) (follow-up: range 2 weeks to 12 weeks)**												

Certainty assessment							№ of patients		Effect		Certainty	Importance
№ of studies	Study design	Risk of bias	Inconsistency	Indirectness	Imprecision	Other considerations	[Intervention]	[Comparison]	Relative (95% CI)	Absolute (95% CI)		
15	Randomised trials	serious[n]	not serious	not serious	not serious	none	415	367	-	**SMD 0.56 SD lower** (0.74 lower to 0.39 lower)	⊕⊕⊕◯ **Moderate[n]**	CRITICAL
**Short duration (Disability) (follow-up: range 2 weeks to 4 weeks)**												
6	Randomised trials	serious[o]	not serious	not serious	serious[p]	none	179	137	-	**SMD 0.46 SD lower** (0.74 lower to 0.19 lower)	⊕⊕◯◯ **Low[o,p]**	CRITICAL
**Median duration (Disability) (follow-up: median 6 weeks)**												
4	Randomised trials	not serious	not serious	not serious	serious[q]	none	143	140	-	**SMD 0.46 SD lower** (0.75 lower to 0.18 lower)	⊕⊕⊕◯ **Moderate[q]**	CRITICAL
**Maximum duration (Disability) (follow-up: range 8 weeks to 12 weeks)**												
5	Randomised trials	not serious	not serious	not serious	serious[r]	strong association	93	90	-	**SMD 0.85 SD lower** (1.16 lower to 0.53 lower)	⊕⊕⊕⊕ **High[r]**	CRITICAL
**Specific LBP (Disability)**												
8	Randomised trials	serious[s]	not serious	not serious	not serious	none	255	209	-	**SMD 0.42 SD lower** (0.61 lower to 0.23 lower)	⊕⊕⊕◯ **Moderate[s]**	CRITICAL
**Non specific (Disability)**												
7	Randomised trials	not serious	not serious	not serious	serious[t]	none	160	158	-	**SMD 0.73 SD lower** (0.96 lower to 0.5 lower)	⊕⊕⊕◯ **Moderate[t]**	CRITICAL
**Core stability exercises (Disability)**												

Certainty assessment							№ of patients		Effect		Certainty	Importance
№ of studies	Study design	Risk of bias	Inconsistency	Indirectness	Imprecision	Other considerations	[Intervention]	[Comparison]	Relative (95% CI)	Absolute (95% CI)		
6	Randomised trials	serious[u]	not serious	not serious	serious[v]	none	129	128	-	SMD **0.62 SD lower** (0.88 lower to 0.37 lower)	⊕⊕◯◯ **Low**[u,v]	CRITICAL
**Spinal stability exercises (Disability)**												
9	Randomised trials	not serious	not serious	not serious	not serious	none	286	239	-	SMD **0.56 SD lower** (0.79 lower to 0.32 lower)	⊕⊕⊕⊕ **High**	CRITICAL
**Low risk (Disability)**												
8	Randomised trials	not serious	not serious	not serious	not serious	none	210	210	-	SMD **0.63 SD lower** (0.83 lower to 0.44 lower)	⊕⊕⊕⊕ **High**	CRITICAL
**High risk (Disability)**												
7	Randomised trials	very serious[w]	not serious	not serious	serious[x]	none	205	157	-	SMD **0.5 SD lower** (0.81 higher to 0.18 higher)	⊕◯◯◯ **Very low**[w,x]	IMPORTANT

CI: confidence interval; **SMD:** standardised mean difference. **Explanations:** a. Seven high-risk studies. b. 50% of high-risk studies. c. Less than 400 participants. d. Less than 400 participants. e. Two high-risk studies. f. Less than 400 participants. g. Less than 400 participants. h. 50% of high-risk studies. i. Less than 400 participants. j. Less than 400 participants. k. Only 74 respondents participated. l. All studies had high risk. m. Less than 400 participants. n. Seven high-risk studies. o. Four high-risk studies p. Less than 400 participants. q. Less than 400 participants. r. Less than 400 participants. s. 50% of high-risk studies. t. Less than 400 participants. u. 50% of high-risk studies. v. Less than 400 participants. w. All studies have high risk. x. Less than 400 participants.
